# A pilot study of dronabinol for the treatment of pain in sickle cell disease

**DOI:** 10.1186/s40814-025-01705-6

**Published:** 2025-11-12

**Authors:** Susanna A. Curtis, Ritika Jhawar, Jordan Bellis, Sarah McCuskee, Lesley Devine, John D. Roberts

**Affiliations:** 1 Department of Medicine, Yale University, New Haven, CT USA; 2 Department of Emergency Medicine, Icahn School of Medicine at Mount Sinai, New York, NY USA

**Keywords:** Sickle cell disease, Dronabinol, Chronic pain, Cannabinoids, Feasibility

## Abstract

**Background:**

Many adults living with sickle cell disease (SCD) suffer from chronic pain, turning to cannabis for relief. However, there are few studies that examine the efficacy of cannabis in treating pain associated with SCD. Prior to an efficacy study, a feasibility study is necessary to evaluate if such a study would be acceptable to patients, including those who use cannabis; if participants would be able to abstain from other cannabinoid-containing substances during the study; if masking would be feasible; and if dronabinol would prove safe.

**Methods:**

We approached outpatients with SCD of any genotype at healthy baseline and asked about interest in such a study. If eligible enrolled participants received dronabinol or placebo for two, 2-week periods. Feasibility outcomes were acceptability (patient interest and enrollment rates), protocol adherence (completion of stud procedures), cannabinoid avoidance, and masking effectiveness. Patient-reported outcomes (PRO), laboratory markers of inflammation, and urine tests for the presence of cannabinoids were collected after each exposure period.

**Results:**

A total of 27 patients were approached; 23 (85%) were interested, 13 (48%) signed consents, and 6 (22%) were enrolled so the study was determined to be acceptable. Patients who used unregulated cannabis and medical cannabis also found the study acceptable. All enrolled participants successfully completed all study procedures. Urine testing revealed no cannabinoid use except for study drug, so the study was determined to be feasible. While 4 out of 6 (67%) participants correctly identified their exposure assignment after the first study period, all 6 (100%) identified the exposure assignment after the second treatment period, so masking after the second period was not feasible. No serious adverse events were attributed to dronabinol.

**Conclusion:**

In conclusion, a controlled study of dronabinol is acceptable, feasible, and safe to participants. However, a crossover design compromises participant masking. A larger, longer, controlled efficacy study without the crossover component is now being performed (NCT05519111).

Trial registration

NCT03978156. Date of registration: 07/26/2019.

## Key messages regarding feasibility

- What uncertainties existed regarding the feasibility? We sought to study whether dronabinol, an FDA-approved oral cannabinoid, would ameliorate pain in people living with sickle cell disease in a randomized placebo-controlled crossover study. However, before an efficacy study could be done, key feasibility questions had to be answered. 1. Would patients living with sickle cell disease be interested in participating in a study of an FDA-approved cannabinoid, especially those already using cannabis containing products as both unregulated cannabis use for the treatment of pain is common in this population and we certify patients for medical cannabis at our center. 2. Would patients who were already using cannabis be willing and able to avoid all other cannabinoid-containing products while on study? 3. Will the psychoactive components of dronabinol enable subjects to be able to distinguish dronabinol from placebo?
- What are the key feasibility findings? 1. Patients living with sickle cell disease are interested in participating in a study of dronabinol for the treatment of pain, even those who use unregulated cannabis or medical cannabis. 2. Subjects are willing and able to avoid all other cannabinoid-containing products. 3. While only 4/6 subjects guessed study assignment in the first treatment phase, 6/6 guessed assignments of both phases in the second phase which suggests the crossover design of the study makes masking dronabinol more difficult.
- What are the implications of the feasibility findings for the design of the main study? A longer, larger, phase 2 study for examining the efficacy of dronabinol for the treatment of pain in people living with sickle cell disease is now being performed (NCT05519111); however, the design is no longer a crossover study given the poor effects on masking.

### Background

Pain is the most common symptom associated with sickle cell disease (SCD) [1]. Smith et al. (2008) reported that 54% of adults with SCD reported pain on most days, and 29% have pain almost daily [21]. Chronic pain defined as pain on most days is multifactorial and can be due to tissue and nerve damage, inflammation, and central and peripheral pain sensitization. A significant minority of adults with SCD, ranging from 25 to 50%, report use of cannabis to treat this pain [2]. Yet, there is only one rigorous study of cannabinoids in SCD, largely due to scientific and regulatory barriers. This was a single-center, prospective, placebo-controlled, double-masked, crossover, randomized study of daily exposure to a pharmaceutical-grade formulation of inhaled cannabis for 5 days [3]. Although improvement in pain did not meet statistical significance, there was an improvement in mood. Other studies have been survey measures of patient-reported use of illicit or, where available, medical or recreational marijuana products [2, 4–7]. These survey studies are heterogeneous in terms of the nature of the cannabinoid (natural or refined product), dose and content of cannabinoids (tetrahydrocannabinol (THC), cannabidiol (CBD/CBG), other), and method of use (inhaled, swallowed, transcutaneous absorption). We hypothesized that these barriers might be addressed by conducting a study using an already FDA-approved cannabinoid. An FDA-approved drug would be pharmaceutical grade, allowing consistency in cannabinoid content, dose, and method of use. There are currently three cannabinoids that exist in FDA-approved form: dronabinol (an oral synthetic THC approved for treatment of nausea and appetite stimulation), nabilone (an oral synthetic analog of THC approved for treatment of nausea), and Epidiolex (a liquid formulation of CBD approved for the treatment of seizure disorders) [8, 9]. We chose to utilize dronabinol, as there is stronger evidence of the effectiveness of THC for the treatment of pain and there is evidence specifically for dronabinol, although CBD has been shown to have possible benefits as well.

A rigorous randomized controlled pilot study examining the possible effect of a cannabinoid treating SCD-related pain should involve prolonged administration and be sufficiently powered to show any clinically significant benefits. However, before such a study is conducted, a pilot study should be done to address four important questions: (1) Would patients with SCD want to participate in such a study? (2) Would patients already utilizing regulated or unregulated cannabis products be interested, would they be willing and able to stop this use while on study, and could this be verified in a quantitative way? (3) Would the psychoactive properties of THC interfere with masking? (4) Would cannabinoid use be safe in people with SCD, particularly with patients using large doses of opioids, which have many adverse effects in common with THC?

We conducted this pilot study to determine the feasibility and acceptability of a study on the use of dronabinol for the treatment of pain in adults with SCD. Feasibility was defined as the percent of enrolled patients who completed all study procedures. Our study was a randomized, placebo-controlled, double-masked, crossover pilot study involving 2 weeks of patient-optimized doses of dronabinol/placebo followed by a 2-week wash-out period and concluding in 2 weeks of whichever treatment was not previously received. Our goal was to confirm that patients living with SCD would be interested in such a study, that patients who are already utilizing regulated or unregulated products would be interested, that masking could be preserved, and that such a study would be safe. While small pilot studies cannot prove the efficacy of a treatment, they can optimize study design and improve the odds of launching and successfully pursuing a larger, more expensive, definitive study with appropriate endpoints.

### Methods

The pilot protocol was approved by the Yale University Institutional Review Board. All participants were provided with written informed consent before study enrollment. The study was performed, and the data was collected at the Sickle Cell Program at the Yale Cancer Center. The trial was registered at clinicaltrials.gov NCT03978156.

### Patients

Inclusion criteria: Any genotype of SCD (homozygous hemoglobin S (HbSS), sickle hemoglobin C disease (HbSC), sickle β^0^ thalassemia (HSβ^0^), sickle β^+^ thalassemia (HSβ^+^), or other genotypes), adults (≥ 18 years of age), pain at baseline (defined by Adult Sickle Cell Quality of Life Measurement Information System (ASCQ-Me) Pain Impact score ≤ 60), urine toxicology test negative for cannabinoids within 30 days of signing consent, stable dose of any disease-modifying medication (hydroxyurea, L-glutamine, or scheduled blood transfusions) for 3 months.

#### Exclusion criteria

Previous diagnosis of psychosis, pregnancy, or breastfeeding, and ability to become pregnant with unwillingness to use acceptable contraception.

#### Trial design

**Fig. 1:**
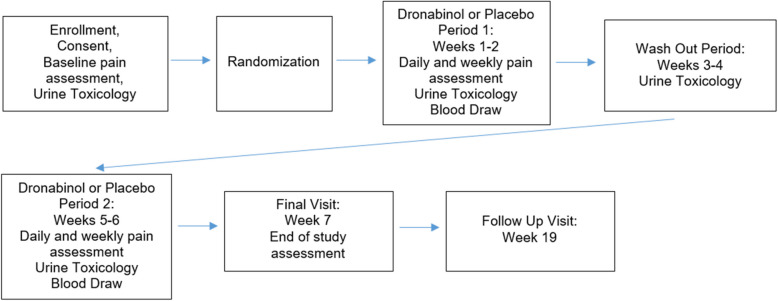
Study scheme

This study was a randomized, placebo-controlled, double-masked crossover feasibility study. The pilot study consisted of a screening phase, a 7-week long treatment period, and a 12-week follow-up evaluation phase. The treatment period consisted of two 2-week treatment phases with a 2-week wash-out period in between and a follow-up visit after all treatment has been completed on the seventh week (Fig. 1). Participants were enrolled during clinic visits for regular care at the Sickle Cell Center at the Yale Cancer Center. Participants were randomized to receive either placebo or dronabinol first in blocks of four, and randomization was performed centrally without stratification. Randomization and participant assignment were done electronically by the investigational pharmacist. Enrollment was done by the investigators.

#### Dose

**Fig. 2:**
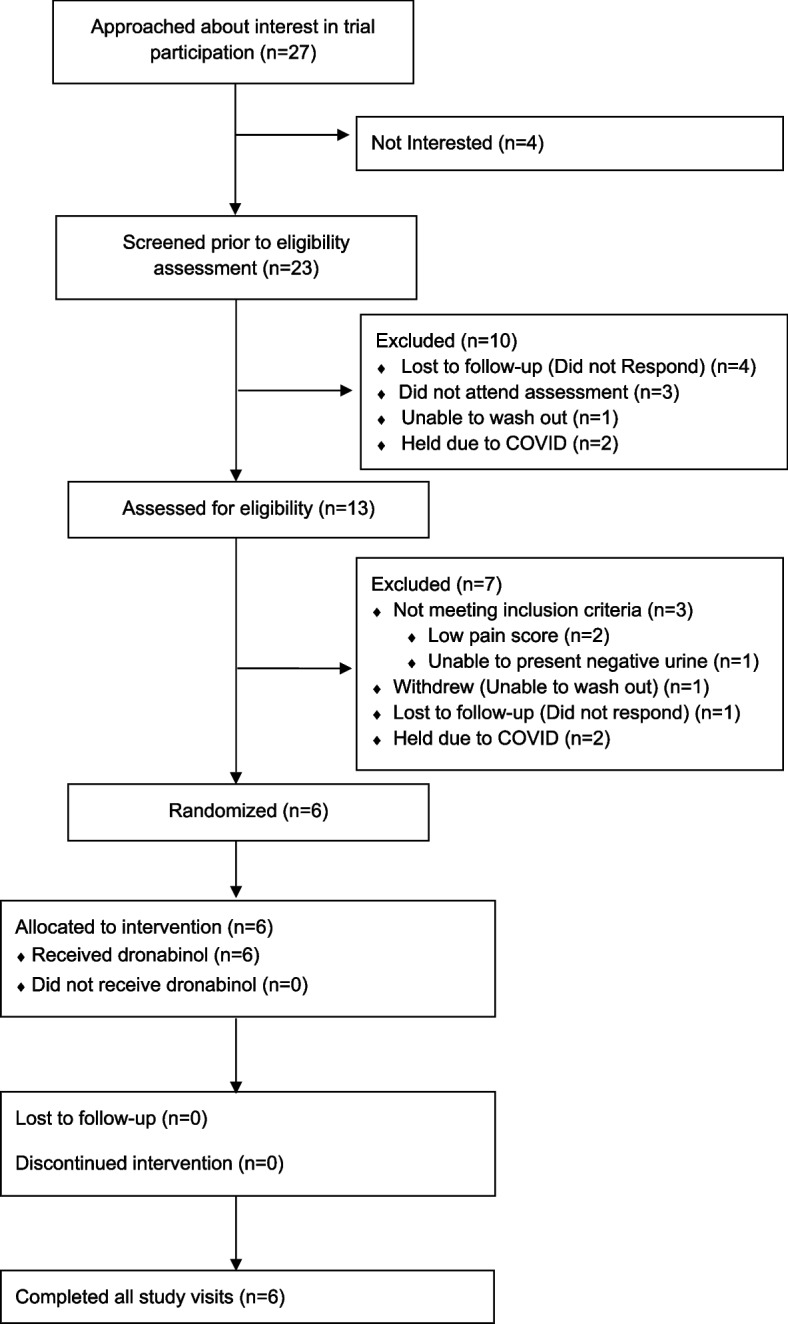
CONSORT statement

The dose of dronabinol was titrated from 2.5 mg twice daily to 10 mg twice daily to suit each patient individually. Participants initially took a dose of 5 mg twice daily on the first day of treatment. Investigators then made daily phone calls during the next 4 days to determine the individualized dose. The goal of titration was to reach the maximum dose at which no unpleasant side effects were experienced by the patient. Participants then continued with their individualized dose for the remainder of the treatment phase (Fig. 2).

#### Outcomes

The primary outcomes of this pilot study were feasibility and acceptability. Acceptability was defined as the percentage of patients who expressed interest in the study, the percentage of those who signed consent, and the percentage of randomized participants. Feasibility was determined by the percentage of study procedures completed by randomized participants (including weekly visits, weekly surveys, pill diaries, lab draws, and urine toxicology tests), the percentage of participants who refrained from using other cannabinoids during the study period, and the success of study masking.

We determined based on other pilot studies examining acceptability and feasibility that the study would be considered acceptable to either the general population or people who already utilized cannabis if > 70% of patients expressed interest in the study. Acceptability would further require that 50% of patients who reported interest in the study were willing to return for screening and if 50% of those patients were randomized and enrolled as we estimated the other 50% of patients may not meet eligibility criteria.

If the study was not acceptable to the general population, we would not move forward with the efficacy study. If the study was not acceptable to those that already utilize related or unregulated cannabis, they would be excluded from the efficacy study.

We determined based on previous pilot studies that the study would be considered feasible if enrolled patients attended 90% of study visits and completed 90% of study procedures and if < 20% of patients had evidence of other cannabinoid use during the study period. If the study was not feasible based on these criteria, we would not move forward with the efficacy study.

We determined that masking would be considered successful if at each timepoint < 75% of patients were unable to correctly guess their treatment assignment. If masking was not successful based on this criterion, we would consider other methods for masking.

We would consider dronabinol unsafe in this population if more than 1 severe adverse event was attributed to dronabinol.

The study’s acceptability was initially assessed by querying all participants about their history of prior cannabis and medical cannabis use. History of prior cannabis use was determined by participants’ self-report of previous lifetime cannabis use. History of medical cannabis use was defined as the dispensation of a medical marijuana product recorded within at least 3 months of study enrollment.

Avoidance of other cannabinoids: Urine samples were collected at the end of each treatment phase and at the end of the wash-out phase. These samples were tested for metabolites of multiple cannabinoids including THC (THC-COOH), CBD, and CBG. Since cannabinoids derived from plants consist of multiple cannabinoids, while dronabinol consists of THC alone, the presence of non-THC cannabinoid metabolites was considered to be indicative of use of cannabinoid products other than dronabinol. In a previous publication, we demonstrated that the presence of CBD and CBG in the urine was 96.4% sensitive in differentiating the use of other cannabinoids from dronabinol alone [10]. Cannabinoids were detected using multiple reaction monitoring using a Xevo TQ-S triple quadrupole mass spectrometer (Waters), operated in negative electrospray ionization mode [10]. Investigators remained masked to the results of all urine testing to preserve study masking.

Study masking: Both participants and investigators were masked throughout the study. Participants were asked whether they believed they were receiving dronabinol or placebo at the end of each treatment phase. At the follow-up study visit, they were then asked during which phase they thought they had received dronabinol and during which phase they thought they had received placebo.

### Secondary outcomes

#### Patient characteristics

Patient characteristics collected included age, gender, race, ethnicity, SCD genotype, medications, history of cannabis use, and history of medical cannabis use. SCD severity was collected using the ASCQ-Me sickle cell disease medical history checklist (SCD-MHC) [11, 12]. ASCQ-Me is a patient-reported outcome survey system comprising multiple quality of life domains validated for adults with SCD. In the ASCQ-Me validation cohort, patients who self-reported excellent health had an average SCD-MHC score of 1, those reporting good health had an average score of 1.9, and those reporting poor health had an average score of 2.6.

#### Patient-reported outcomes

Patient-reported outcomes (PRO) were collected using ASCQ-Me surveys. The ASCQ-Me surveys were chosen as they are previously validated publicly accessible surveys developed by the National Institutes of Health using people living with SCD as the anchoring population, so they were most relevant for our population. For any PRO for which no ASCQ-Me survey existed, PROMIS was used, as these are also previously validated publicly accessible surveys developed by the National Institutes of Health, though the anchoring population varies depending on the chosen survey.

#### Pain-related secondary outcomes

The secondary outcomes related to pain included comparing the ASCQ-Me domain for pain interference at the end of the last week of each treatment phase. Median ASCQ-Me scores for each domain in the validation cohort were 50 with standard deviations of 10. Lower scores represent more severe symptoms, and a difference of 5 points was considered clinically meaningful. All ASCQ-Me scores assessed symptoms experienced in the past 7 days. Pain-related outcomes also included the Patient-Reported Outcomes Measurement Information System (PROMIS) domains for nociceptive pain quality and neuropathic pain quality. PROMIS is a patient-reported outcome survey system validated in the general population [13–15]. All PROMIS scores also assessed symptoms in the past 7 days. Median PROMIS scores for each domain were 50, with standard deviations of 10, where lower scores represent less severe symptoms.

Quality of life-related secondary outcomes: Secondary measures of quality of life included the ASCQ-Me domains for stiffness, emotional interference, and sleep interference, as well as the PROMIS domains for anxiety and gastrointestinal distress, nausea, and vomiting.

#### Laboratory markers

Laboratory tests included baseline safety assessments, which were collected before randomization, unless they had been obtained for clinical purposes within the previous 90 days. These assessments encompassed a comprehensive metabolic panel, liver function tests, magnesium, uric acid, lactate dehydrogenase, and complete blood count with reticulocyte count. All safety assessments were processed at the central hospital laboratory and repeated at the end of each treatment phase. Additional markers of inflammation were assessed, including C-reactive protein and tryptase, and processed at the central hospital laboratory. ELISA for substance P was performed in a shared resource facility, and a cytokine panel (Il1b, Il1a, Il4, Il6, Il8, TNFα, and INFγ) was analyzed using Luminex 200.

#### Safety

Safety assessments were conducted during the screening phase, weekly throughout each treatment phase, at the final treatment visit, and during the follow-up evaluation. These assessments comprised physical examinations, vital sign measurements, and clinical laboratory tests. Any reported adverse events were coded using the NCI Common Terminology Criteria for Adverse Events (CTCAE) v5.0. Additionally, an independent data and safety monitoring committee reviewed safety throughout the pilot study.

Confidentiality and data management: Confidentiality was maintained by storing all data without patient identifiers. Data was stored in a locked office and will be shared with investigators upon request.

#### Statistical analysis

As this was a pilot study and not an efficacy study, power calculations were not conducted, and participants were enrolled as a convenience sample. Patient characteristics and the correlation of these characteristics with study interest and enrollment were examined using descriptive statistics. Means and 95% confidence intervals (CI) were calculated for data with a normal distribution, while medians and interquartile ranges (IQR) were used for non-normally distributed data. Comparisons of study acceptability between non-users of cannabis, those with a history of cannabis use, and medical cannabis users were made using the Kruskal–Wallis test; a *p* value of < 0.05 was considered statistically significant.

#### Sample size

We estimated that if we approach 50 patients in 18 months, 80% would endorse interest in the study and that 25–50% of the patients approached (12–25 patients) would be either regulated or unregulated cannabis users and this would allow us to assess our acceptability criteria. We estimated that if we approached 50 patients in 12 months, 50% of those who endorsed interest would agree to return for screening (20 patients) and half of those would be eligible for the study and would be randomized (10 patients) and this would be sufficient to determine if the study is feasible and if masking is feasible.

## Results

**Fig. 3:**
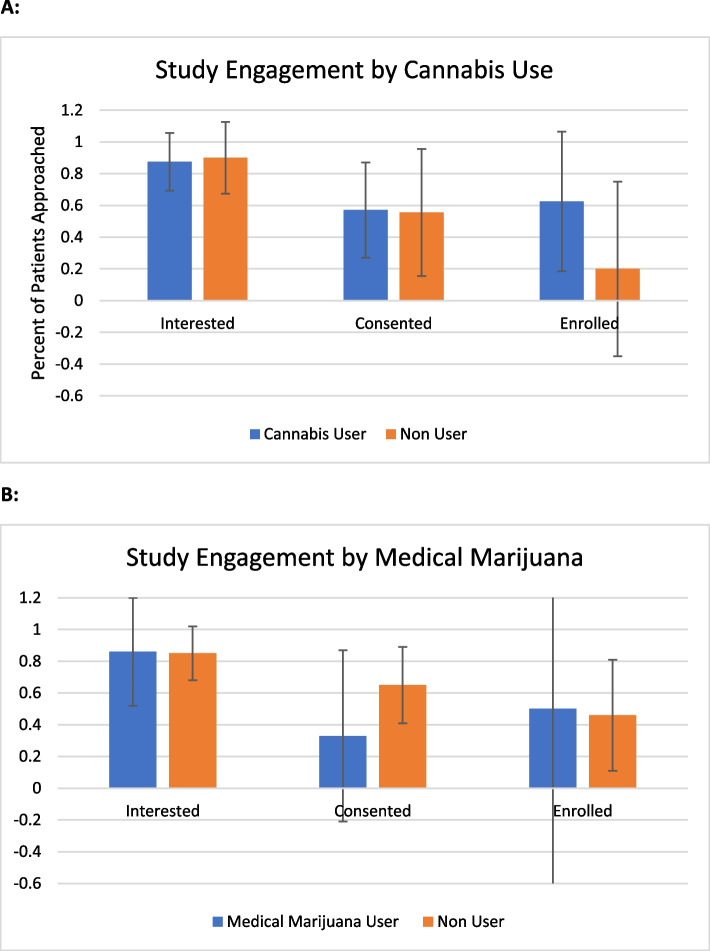
Study engagement stratified by previous cannabis and current medical marijuana use. Percent (with 95% confidence interval) of patients approached for the study who were interested, who consented, and who were enrolled by **A** previous cannabis use and **B** medical marijuana use. All *p* values ≤ 0.05

The study began recruitment in July 2019 and was open until March 2020. Recruitment had to halt prematurely due to the COVID-19 pandemic. During the recruitment period, 27 patients were approached and asked if they would be interested in enrolling in a double-masked study of dronabinol for the treatment of pain in sickle cell disease. Of these, 23 out of 27 (85.2%, 95% CI 66.3–96.8) expressed interest. Of those not interested, one declined participation in any research, one wished to optimize their medical care before participating in research, and two sought parental permission prior to enrolling in any research (Fig. 3, CONSORT statement). Of those who expressed interest, 13 signed consent and were assessed for eligibility (56.5%, 95% CI 34.5–76.8). Among those who expressed interest but did not consent, four were lost to future follow-up, three scheduled consent appointments but did not attend, one attempted but was ultimately unsuccessful with abstaining from cannabis use prior to the consent appointment, and two had their consent appointments canceled due to the COVID-19 pandemic. While the study was stopped early, criteria for acceptability to the general population was > 70% so was considered met.

Of the 13 participants who consented for the study, 6 out of 13 (46.2% 95% CI 19.2–74.9) were found eligible and enrolled. Among those found ineligible, two had pain interference scores insufficient for enrollment within the previous 7 days, one was lost to follow-up after consent, one withdrew due to inability to abstain from cannabis use, one was unable to produce a urine toxicology study that was negative for cannabis, and two had all future study visits canceled due to the COVID-19 pandemic. Notably, all 3 participants unable to abstain from cannabis use or provide a negative urine sample were daily cannabis users. While the study was stopped early, criteria for acceptability was considered met, as ~ 50% of those who expressed interest consented for the study and returned for screening and ~ 50% of those patients were eligible for the study and were enrolled.

Of the 6 participants deemed eligible for the study, 100% were randomized and completed all study visits. One patient had one visit converted to a telehealth visit due to the COVID-19 pandemic and thus, the scheduled blood and urine samples were unable to be collected. No enrolled participants were lost to follow-up, and none withdrew from the study. When adherence to cannabis avoidance was examined, 6 out of 6 participants had THC but no other cannabinoids present in their urine during their active treatment block, while 6 out of 6 participants had no cannabinoids present in their urine during both their placebo treatment block and the wash-out block, confirming that adherence to cannabis avoidance was 100%. The study was considered feasible as > 90% of study procedures were completed and < 20% of subjects have evidence of other cannabis use during the study period.

Among patients approached about the study, 16 out of 27 had a history of lifetime cannabis use, and 7 out of 27 were certified for medical cannabis. Neither lifetime cannabis use nor medical cannabis certification was associated with interest in the study, likelihood of consent, or enrollment. Lifetime cannabis users: interest (87.5%, 95% CI 61.7–98.5), consent (57.1%, 95% CI 28.9–82.4), enrollment (62.5%, 95% CI 24.5–91.5). Medical cannabis users: interest (85.7%, 95% CI 42.1–99.6), consent (33.3%, 95% CI 4.3–77.7), enrollment (35.4%, 95%CI 12.5–98.7). As more than 70% of patients with either a lifetime history of cannabis use and/or medical cannabis use expressed interest in the study, the study was considered acceptable to this population as well.

Regarding the effectiveness of masking, it was found that 4 out of 6 participants correctly identified their treatment assignment after the first treatment block (66.6%). However, all 6 participants (100%) correctly identified their treatment assignment during the second block. Additionally, when asked at the end-of-study visit during which block they received each treatment, all 6 participants (100%) correctly identified each study block. As > 75% of patients were able to guess their assignment during the second block and during both blocks after the second block had taken place, it was determined that the presence of the second block or the crossover design of the study made the masking not feasible in this study.

The enrolled participants had a median age of 26.5 years (range 21–44 years), with 50% being HbSS/HbSβ0 and 50% being HbSC/HbSβ + (Table 1). When patient-reported outcomes (PROs) were examined, pain impact showed improvement by a mean of 3.5 (95% CI −3.53–10.53). Other PROs and changes in laboratory markers were also reported (Table 2).

No severe adverse reactions were attributed to dronabinol. Therefore, dronabinol was considered safe enough in this population to proceed with a future efficacy study.

**Table 1:** Clinical characteristics of patients enrolled in the study

Patient	Gender	Age	Race	SCD genotype	SCD-MHC score	Previous cannabis use	Medical marijuana use
1	M	21	Black or African American	HbSS	5	Yes	No
2	F	44	Black or African American	HbSC	2	Yes	No
3	M	22	Black or African American	HbSβ^+^	1	Yes	Yes
4	F	32	Black or African American	HbSS	4	Yes	No
5	F	28	Black or African American	HbSC	1	No	No
6	F	25	Black or African American	HbSS	3	Yes	No

**Table 2:** Change in PRO scores, laboratory measures, and cytokines from placebo period to active treatment period

	**Mean difference**	**95% confidence interval**
**PROs**		
**Higher is better**		
Pain impact	3.5	[−3.53, 10.53]
Stiffness	0.33	[−9.58, 10.25]
Sleep	4.83	[−3.69, 13.35]
Emotional impact	0.17	[−4.54, 4.87]
**Lower is better**		
Neuropathic quality	−6	[−18.33, 6.33]
Nociceptive quality	−6.52	[−17.69, 4.66]
GI distress	−0.17	[−3.18, 2.84]
Anxiety	4.5	[−2.11, 11.11]
**Laboratory measures**		
Hemoglobin (g/dL)	0.23	[−0.30, 0.76]
White blood count (× 10^3^/µL)	0.23	[−2.22, 2.69]
Platelets (× 10^3^/µL)	−19.33	[−52.69, 14.03]
Absolute neutrophils (× 10^3^/µL)	1.2	[−0.74, 3.14]
Absolute lymphocytes (× 10^3^/µL)	−1.0	[−1.83, −0.17]
C-reactive protein (mg/L)	−0.16	[−1.82, 1.50]
Lactate dehydrogenase (U/L)	−24.4	[−44.58, −4.22]
IL1a (pg/ml)	−0.68	[−2.88, 1.52]
IL1b (pg/ml)	−0.34	[−1.01, 0.33]
IL4 (pg/ml)	−28.36	[−85.53, 28.81]
IL6 (pg/ml)	7.11E−16	[−0.74, 0.74]
IL8 (pg/ml)	2.54	[−1.57, 6.65]
TNFα (pg/ml)	4.22	[−1.54, 9.98]
INFγ (pg/ml)	4.20	[−7.15, 15.55]

*PRO* patient-reported outcome. For all PROs, median is 50; standard deviation is 10. For higher is better, an increase in score represents an improvement in symptoms. For lower is better, a decrease in score represents an improvement in symptoms

## Discussion

This pilot study was designed not to test the efficacy of dronabinol for pain in SCD, but to determine the feasibility and acceptability of a future larger efficacy study. A randomized, placebo-controlled, double-masked, crossover study of dronabinol for the treatment of pain in SCD was found to be acceptable to patients regardless of their history of illicit or medical cannabis use. The study was feasible, as all participants abstained from other cannabinoid-containing products and completed all study visits. Feasibility was determined based on achievement of pre-specified targets including target recruitment rates (> 70% patient interest), protocol adherence (> 90% completion of procedures), and avoidance of other cannabinoids (< 20% evidence of non-study cannabinoid use). Our study met all of these feasibility criteria. However, all participants correctly identified their treatment assignment after the crossover portion of the study, suggesting that crossing over may be detrimental to study masking. Future studies aimed at evaluating the possible efficacy of cannabinoids for chronic pain should consider that people living with SCD are interested in such studies and that these studies can be conducted in a rigorous manner even in participants with a history of prior or active cannabis use. However, crossover designs should be avoided to preserve masking.

A unique challenge in studying cannabinoids is their easy accessibility to patients, for both medicinal and recreational purposes [16]. There are over 120 identified cannabinoids, each with differing effects on varying neuroreceptors which can be modulated by mode of ingestion and presence of other cannabinoids [17]. Therefore, to rigorously examine the effects of a cannabinoid in any population, it is vital to ensure that participants are not taking other cannabinoid-containing substances during the study and that any previously used cannabinoids have been washed out before starting the study. To ensure wash-out, we required each patient to present a urine sample negative for cannabinoids prior to randomization. Multiple participants interested in enrollment who reported cannabis use in the recent past were advised to abstain and return in 3 to 4 weeks when their urine was likely to be negative for cannabinoids. This proved to be a barrier to enrollment for three participants, all of whom were daily cannabis users. While other current cannabis users and even medical cannabis users consented and were enrolled in the study, none of them were daily users. Previous studies have shown that people with SCD who use cannabis primarily utilize it to treat symptoms of their disease, most often pain [4–7]. We hypothesize that daily users were unable to abstain from cannabis use because it was a significant part of their treatment regimen, and abstention subjected them to intolerable worsening of their symptoms. Enrolled participants had urine results showing no other cannabinoid use throughout the study, illustrating that current cannabis users other than daily users were able to avoid other cannabinoids during the study [10]. We concluded that daily cannabis use should be an exclusion criterion in future studies. We also concluded that urine screening for multiple cannabinoids should be done in future studies to identify any participants who utilize non-study cannabinoids, although this is likely to be rare.

A second challenge in studying cannabinoids is their psychoactive nature, which can interfere with masking [18, 19]. Some researchers have suggested replacing placebos in cannabinoid studies with active substances that would cause dry mouth or tachycardia [18, 19]. However, we decided that since the psychoactive effects of cannabinoids are highly dose-dependent and we intended to administer individualized doses, we would first examine the effectiveness of our masking. We found that during the initial treatment period < 75% of participants were able to correctly identify their treatment assignment. However, after the crossover, all participants correctly identified their treatment assignment during both periods. Based on this, we concluded that we should eliminate the crossover portion of the study, as it interfered with masking, but that dronabinol may be adequately masked with placebo alone in a non-crossover study as < 75% of patients correctly guessed their assignment in the first block.

### Strengths and limitations

Our study serves as a pilot study aimed at optimizing the design of a future efficacy study. While participants in our study demonstrated a mean improvement in their pain impact score and a reduction in mean white blood cell count (Table 2, Fig. 3), neither of these findings reached statistical significance, likely due to being underpowered to examine these outcomes. Larger studies would be required to determine if dronabinol is beneficial for pain or other quality of life symptoms, or for reducing inflammation in SCD. Furthermore, chronic pain often has components of neuropathic pain and peripheral and central sensitization. Treatment guidelines for these types of pain recommend an 8-week study of a new medication to determine efficacy. Thus, our study duration is likely too short to optimally examine the effects of dronabinol on pain in SCD [20]. The protocol for the study was not published prior to the pilot study. An implementation framework was not used to determine data collected. Patients and the public were not involved in the design of the pilot study but will be involved in the design of the future efficacy study.

## Conclusions

We showed that studying an FDA-approved oral cannabinoid is acceptable to patients with SCD whether or not they have previously used regulated or unregulated cannabis and that such a study is feasible. However, we did determine that a crossover design makes masking dronabinol unfeasible. Our study serves as a pilot study aimed at optimizing the design of a future efficacy study. While participants in our study demonstrated a mean improvement in their pain impact score and a reduction in mean white blood cell count (Table 2, Fig. 3), these findings were underpowered to examine these outcomes. Larger studies would be required to determine if dronabinol is beneficial for pain or other quality of life symptoms, or for reducing inflammation in SCD. Furthermore, chronic pain often has components of neuropathic pain and peripheral and central sensitization. Treatment guidelines for these types of pain recommend an 8-week study of a new medication to determine efficacy. Thus, our study duration is likely too short to optimally examine the effects of dronabinol on pain in SCD [20].

There is a critical need for rigorous studies of cannabinoids in SCD partly due to the lack of effective treatments for chronic pain, but also because cannabinoid use is common in SCD, and clinicians need to understand the risk and benefits of this use to advise patients. Based on the findings of our pilot study, we designed a randomized, placebo-controlled, double-masked study of 8 weeks of dronabinol for the treatment of chronic pain and/or reduction of inflammation in SCD without a crossover component. This study is now open for enrollment (NCT05519111). Studies of other cannabinoids, such as CBD and other modes of use such as mucosal or topical agents, should also be conducted.

## Data Availability

The datasets during and/or analyzed during the current study are available from the corresponding author on reasonable request.
